# Enantioselective Michael Addition of 3-Aryl-Substituted Oxindoles to Methyl Vinyl Ketone Catalyzed by a Binaphthyl-Modified Bifunctional Organocatalyst

**DOI:** 10.3390/molecules17067523

**Published:** 2012-06-18

**Authors:** Hyun Joo Lee, Saet Byeol Woo, Dae Young Kim

**Affiliations:** Department of Chemistry, Soonchunhyang University, Asan, Chungnam 336-745, Korea

**Keywords:** oxindole, methyl vinyl ketone, conjugate addition, bifunctional organocatalysis, asymmetric catalysis

## Abstract

The enantioselective conjugate addition reaction of 3-aryl-substituted oxindoles with methyl vinyl ketone promoted by binaphthyl-modified bifunctional organocatalysts was investigated. The corresponding Michael adducts, containing a quaternary center at the C3-position of the oxindoles, were generally obtained in high yields with excellent enantioselectivities (up to 91% *ee*).

## 1. Introduction

Oxindole structures exist in a large number of natural and biologically active molecules [1,2,3,4]. In particular, oxindole scaffolds bearing a quaternary stereocenter at the 3-position are a versatile structural motif found in a variety of biologically and pharmaceutically active natural products and utilized as building blocks for indole alkaloid synthesis [5]. Several methods for their asymmetric formation and transformation are of considerable interest. Discovering various electrophiles to react with 3-substituted oxindoles for the synthesis of diversely structured 3,3-disubstituted oxindoles is still strongly desired. Among the established strategies for the synthesis of chiral 3,3-disubstituted oxindoles, a transition metal-catalyzed asymmetric reaction has been intensively studied [6,7,8,9,10,11,12,13,14,15,16,17,18]. Recently, organocatalytic enantioselective conjugate addition reactions of oxindoles with enals, nitroalkenes, and vinyl sulfones have been reported [19,20,21,22,23,24,25,26,27,28,29,30,31,32,33]. Several groups have reported the enantioselective conjugate addition reactions of 3-substituted oxindoles to vinyl ketones catalyzed by organocatalysts, phase-transfer catalyst, and chiral calcium phosphate [34,35,36,37,38]. Although there have been reports on the catalytic enantioselective conjugate addition reaction of 3-substituted oxindoles to vinyl ketones using organocatalysts such as bifunctional tertiary-amine thiourea, chiral primary amine, and proline [35,36,37], there are still some drawbacks in the previously reported procedures, such as high catalyst loading and long reaction time. Therefore, the development of alternative catalysts for the catalytic enantioselective conjugate addition reaction of 3-substituted oxindoles to vinyl ketones would be highly desirable. 

## 2. Results and Discussion

**Figure 1:**
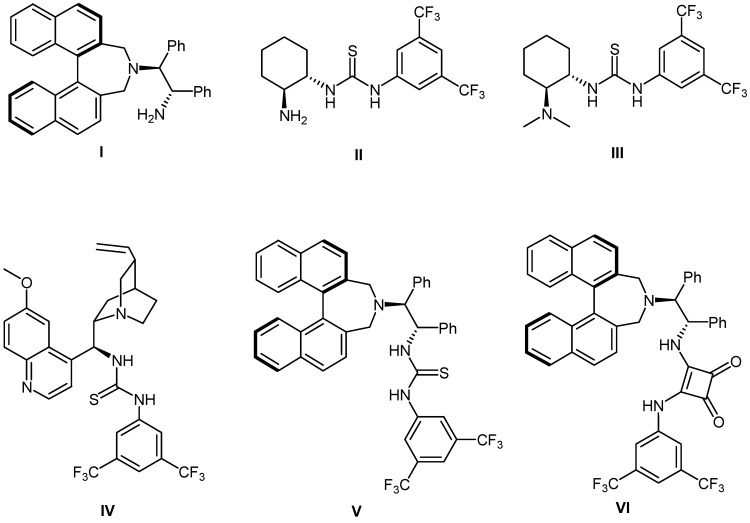
Structures of various chiral organocatalysts.

As part of the research program related to the development of synthetic methods for the catalytic carbon-carbon bond formations [39,40,41,42,43,44,45,46,47,48,49,50], we recently reported the organocatalytic conjugate addition reactions of α,β-unsaturated carbonyl compounds [51,52,53] and other Michael acceptors [54,55,56,57,58,59,60,61,62,63,64,65,66,67,68]. In this Communication, we wish to describe the enantioselective conjugate addition reaction of 3-substituted oxindoles with methyl vinyl ketone catalyzed by binaphthyl-modified bifunctional organocatalysts bearing both central and axial chiral elements. (Figure 1)

In an attempt to validate the feasibility of the organocatalytic enantioselective conjugate addition reaction of 3-substituted oxindoles, we first investigated a reaction system with 3-phenyloxindole (**1a**) with methyl vinyl ketone (**2a**) in the presence of 5 mol% of catalyst in toluene at room temperature. We examined the impact of the structure of catalysts **I–VI** on enantioselectivity (Table 1, entries 1–6) and found that chiral primary amine catalysts **I** and **II** were ineffective (Table 1, entries 1 and 2). In order to enhance the enantioselectivity, bifunctional organocatalysts **III–VI** were evaluated in the model reaction (Table 1, entries 3–6). Takemoto’s catalyst **III** and quinine-derived thiourea catalyst **IV** were less effective (Table 1, entries 3 and 4), whereas the binaphthyl-modified chiral bifunctional organocatalysts **V** and **VI** bearing both central and axial chiral elements, effectively promoted the addition reaction in high yields with high enantioselectivities (Table 1, entries 5 and 6). Further, catalyst **V** gave the desired product **3a** with high enantioselectivity (91% *ee*, Table 1, entry 5). A survey of the reaction media indicated that many common solvents, such as dichloromethane, THF, diethyl ether, and xylene (Table 1, entries 5 and 7–11) were well tolerated in this conjugate addition without significant decreases in enantioselectivity. Among the solvents probed, the best results (88% yield and 91% *ee*) were achieved when the reaction was conducted in toluene (Table 1, entry 5). Catalyst loading down to 2.5 and 1 mol % decreased the enantioselectivity (entries 12 and 13).

**Table 1:** Optimization of the reaction conditions. 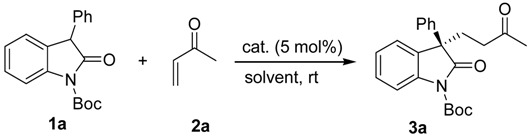

Entry	Cat.	Solvent	Time (h)	Yield (%) ^a^	*ee* (%) ^b^
1	**I**	PhMe	3	73	5
2	**II**	PhMe	3	76	5
3	**III**	PhMe	2	82	25
4	**IV**	PhMe	5	83	15
5	**V**	PhMe	2	88	91
6	**VI**	PhMe	2	80	67
7	**III**	CH_2_Cl_2_	2	90	83
8	**III**	THF	3	78	81
9	**III**	Et_2_O	3	86	73
10	**III**	*p*-xylene	4	78	87
11	**III**	*m*-xylene	4	81	85
12 ^c^	**V**	PhMe	5	83	87
13 ^d^	**V**	PhMe	5	70	59

^a^ Isolated yield; ^b^ Enantiomeric excess was determined by HPLC analysis using Chiralpak AD-H column; ^c^ Reaction carried out using 2.5 mol% of catalyst; ^d^ Reaction carried out using 1 mol% of catalyst.

By employing the optimized reaction conditions in hand, the scope of the methodology was investigated in reactions with 3-aryl-substituted oxindoles **1** and methyl vinyl ketone **2a** in the presence of 5 mol% of binaphthyl-modified thiourea-tertiary amine catalyst **V** in toluene at room temperature (Table 2). A range of electron-donating and electron-withdrawing substitutions on the aryl ring of the 3-aryl-substituted oxindoles **1a–e** provided reaction products in high yields and high enantioselectivities (81–91%, Table 2, entries 1–5). 3-Naphthyl-substituted oxindole **1f** provided product with high selectivity (83% *ee*, Table 2, entry 6). The absolute configuration of **3** was determined by comparison of the optical rotation and chiral HPLC data with the literature values [35,36,37].

**Table 2:** Enantioselective Michael addition of 3-aryl substituted oxindoles **1** to methyl vinyl ketone **2a**. 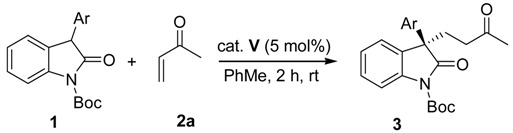

Entry	1, Ar	Yield (%) ^a^	*ee* (%) ^b^
1	**1a**, Ph	88	**3a**, 91
2^c^	**1b**, *m*-MeC_6_H_4_	91	**3b**, 83
3	**1c**, *p*-MeC_6_H_4_	88	**3c**, 81
4	**1d**, *m*-MeOC_6_H_4_	86	**3d**, 91
5 ^c^	**1e**, *p*-FC_6_H_4_	87	**3e**, 85
6	**1f**, 2-naphthyl	82	**3f**, 83

^a^ Isolated yield; ^b^ Enantiomeric excess of 3 was determined by HPLC analysis using Chiralpak AD-H (for **3a**) and IA (for **3b–f**) columns; ^c^ Reaction carried out using 10 mol% of catalyst.

**Scheme 1:**
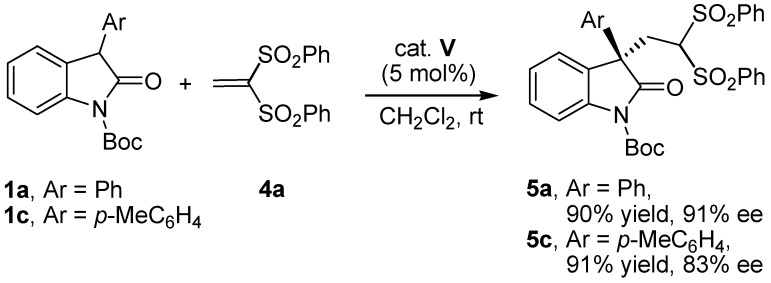
Enantioselective Michael addition of 3-aryl substituted oxindoles **1** to 1,1-bis(benzenesulfonyl)ethylene (**4a**).

In addition to the vinyl ketone **2a**, 1,1-bis(benzensulfonyl)ethylene (**4a**) was also examined in this conjugate addition reaction as a Michael acceptor. The Michael addition reaction of 3-aryl-substituted oxindoles **1** with 1,1-bis(benzensulfonyl)ethylene (**4a**) proceeded to afford the Michael adducts in high enantioselectivities (Scheme 1). Absolute configuration of 5 was determined by comparison of the optical rotation and chiral HPLC data with the literature values [23,24].

## 3. Experimental

### 3.1. General

All commercial reagents and solvents were used without purification. TLC analyses were carried out on pre-coated silica gel plates with F_254_ indicator. Visualization was accomplished by UV light (254 nm), I_2_, *p*-anisaldehyde, ninhydrin, and phosphomolybdic acid solution as an indicator. Purification of reaction products was carried out by flash chromatography using E. Merck silica gel 60 (230–400 mesh). ^1^H-NMR and ^13^C-NMR spectra were recorded on a Bruker AC 200 instrument (200 MHz for ^1^H, 50 MHz for ^13^C). Chemical shift values (δ) are reported in ppm relative to Me_4_Si (δ 0.0 ppm). Optical rotations were measured on a JASCO-DIP-1000 digital polarimeter with a sodium lamp. The enantiomeric excesses (*ee*’*s*) were determined by HPLC. HPLC analysis was performed on Younglin M930 Series and Younglin M720 Series, measured at 254 nm using the indicated chiral column. Mass spectra were obtained on Jeol JMS-DX303 instrument.

### 3.2. Typical Procedure for the Conjugate Addition Reaction of 3-Phenyloxindole (**1a**) with Methyl Vinyl Ketone (**2a**)

To a solution of 3-phenyloxindole (**1a**, 0.3 mmol, 93 mg) and catalyst **III** (0.015 mmol, 11.4 mg) in toluene (1.8 mL) was added methyl vinyl ketone (**2a**, 0.36 mmol, 25 mg). Reaction mixture was stirred for 2 h at room temperature, concentrated, and purified by flash column chromatography (EtOAc-hexane = 1:3) to afford the Michael adduct **3a** (100 mg, 88%).

*(R)-tert-Butyl 2-oxo-3-(3-oxobutyl)-3-phenylindoline-1-carboxylate* (**3a**): [α]^21^_D_= + 43.4 (c = 1, CHCl_3_); ^1^H-NMR (CDCl_3_) δ 7.94 (d, *J* = 8.0 Hz, 1H), 7.42–7.11 (m, 8H), 2.80–2.70 (m, 1H), 2.55–2.28 (m, 2H), 2.12–2.04 (m, 1H), 2.00 (s, 3H), 1.63 (s, 9H); ^13^C-NMR (CDCl_3_) δ 206.5, 176.3, 148.8, 139.7, 139.2, 130.2, 128.5, 127.3, 126.5, 124.5, 124.4, 115.1, 84.5, 55.8, 38.5, 31.4, 29.8, 28.0; HRMS (EI^+^): *m/z* calcd for C_24_H_27_NO_4_ [M]^+^: 393.1940; found 393.1938; HPLC (85:15, *n*-hexane-*i*-PrOH, 254 nm, 1.0 mL/min) Chiralpak AD-H column, t_R_ = 6.85 min (minor), t_R_ = 9.86 min (major), 91% ee.

### 3.3. Typical Procedure for the Conjugate Addition Reaction of 3-Phenyloxindole (**1a**) with 1,1-Bis(benzenesulfonyl)ethylene (**4a**)

To a solution of 3-phenyloxindole (**1**, 0.3 mmol, 93 mg) and catalyst **III** (0.015 mmol, 11.4 mg) in CH_2_Cl_2_ (1.2 mL) was added 1,1-bis(benzenesulfonyl)ethylene (**4a**, 0.45 mmol, 138.7 mg). Reaction mixture was stirred for 2 h at room temperature, concentrated, and purified by flash column chromatography (EtOAc-hexane:1:5) to afford the Michael adduct **5a** (168 mg, 90%).

*(R)-tert-Butyl 3-(2,2-bis(phenylsulfonyl)ethyl)-2-oxo-3-phenylindoline-1-carboxylate* (**5a**): [α]^24^_D_ = 24.8 (c = 0.4, CHCl_3_); ^1^H-NMR (CDCl_3_) δ 8.07–7.97 (m, 3H), 7.77–7.67 (m, 3H), 7.64–7.46 (m, 6H), 7.38–7.16 (m, 7H), 4.45–4.41 (m, 1H), 3.40-3.29 (m, 2H), 1.59 (s, 9H); ^13^C-NMR (CDCl_3_) δ 175.2, 149.2, 141.2, 140.8, 138.0, 134.7, 134.4, 131.0, 129.3, 128.9, 128.8, 128.0, 126.7, 125.7, 124.6, 116.3, 84.3, 80.6, 55.2, 32.1, 28.0; HPLC (90:10, n-hexane-*i*-PrOH, 254 nm, 0.5 mL/min) Chiralcel OD-H column, t_R_ = 18.8 min (major), t_R_ = 23.9 (minor), 91% ee. 

## 4. Conclusions

In conclusion, we have developed a highly efficient catalytic enantioselective conjugate addition reactions of 3-aryl-substituted oxindoles to methyl vinyl ketone using 5 mol% of binaphthyl-modified bifunctional catalyst **V**. The desired Michael products were obtained in good to high yields and enantioselectivities (81–91% *ee*) for the 3-aryloxindoles examined in this work. We believe that this method provides a practical entry for the preparation of synthesis of medicinally useful chiral 3,3-disubstituted oxindoles. Further study of these bifunctional organocatalysts in other asymmetric reactions is being under conducted. 
